# Molecular mechanisms of gut microbiota dysbiosis and metabolites in Alzheimer’s disease pathogenesis: implications for precision therapeutics

**DOI:** 10.1186/s13041-025-01263-1

**Published:** 2025-11-26

**Authors:** Yashar Vaziri, Jaleh Bagheri Hamzyan Olia, Cigir Biray Avci, Alireza Nourazarian

**Affiliations:** 1 Department of Nutrition and Dietetics, Sara.C., Islamic Azad University, Sarab, Iran; 2 Department of Basic Medical Sciences, Khoy University of Medical Sciences, Khoy, Iran; 3 Department of Medical Biology, Faculty of Medicine, Ege University, Izmir, Turkey

**Keywords:** Alzheimer disease, Gastrointestinal microbiome, Microbiota–gut-brain axis, Neuroinflammation, Probiotics, Fecal microbiota transplantation

## Abstract

Alzheimer’s disease (AD) originates from both central and peripheral pathways. The gut microbiota is a clear risk factor. In AD, microbiota imbalances drive immune system activation, disrupt protective barriers, and alter neuromodulatory signaling. Additionally, gut microbiota dysbiosis has been identified as a risk factor for AD. Recent research indicates that dysbiosis of the microbiota in AD is linked to immune activation, barrier dysfunction, and neuromodulatory signaling. Studies of AD pathology reveal that short-chain fatty acids, indole derivatives, and bile acids can have both protective and harmful effects. New strategies, such as probiotics, dietary changes, and fecal microbiota transplantation, may influence disease progression in AD. However, conflicting methods, unaccountable motives, and ethical concerns surrounding microbiome interventions pose significant hurdles. To translate findings related to the gut-brain axis into effective solutions, we need standardized multi-omics approaches, personalized therapies, and oversight from regulatory authorities. Ultimately, leveraging insights from the gut microbiome holds great promise for transforming how we diagnose, prevent, and treat AD.

## Introduction

**Fig. 1:**
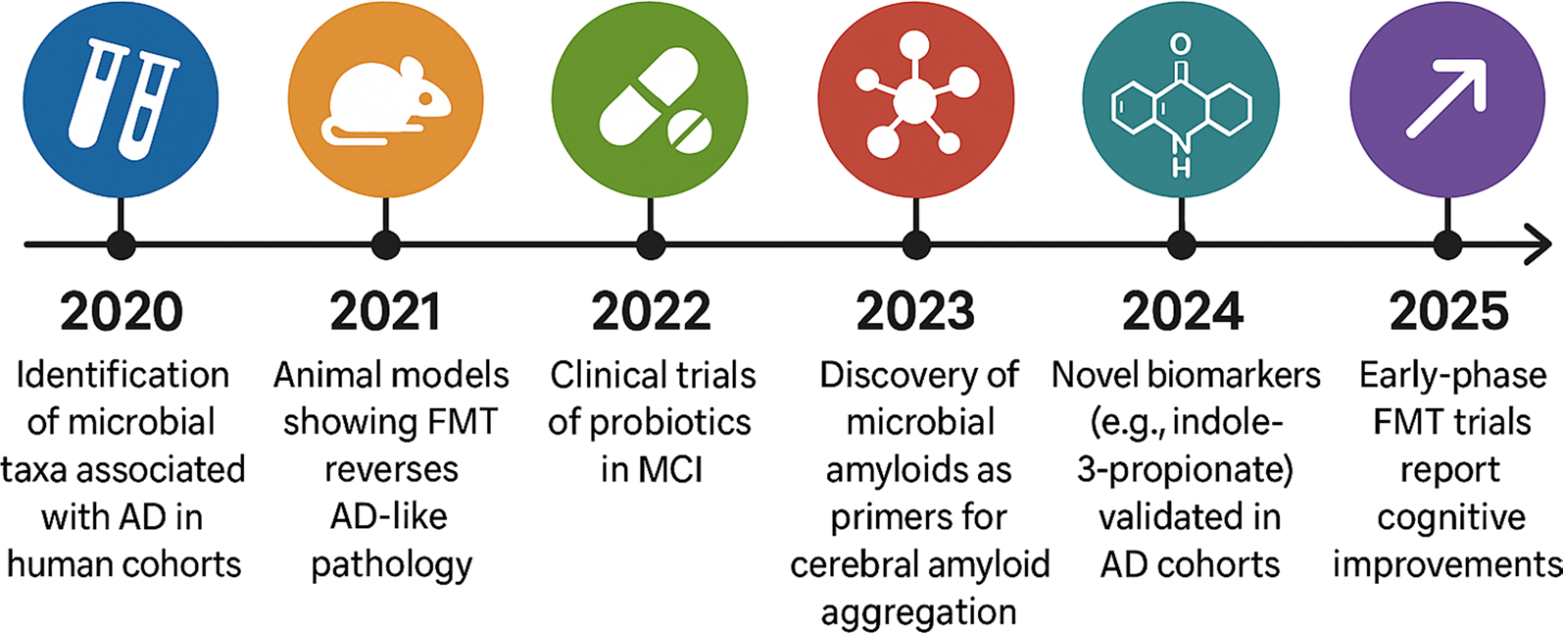
Timeline of key discoveries in gut-brain axis and AD research (2020–2025)

AD is a progressive, irreversible neurodegenerative disorder and the leading cause of dementia. Its cognitive and behavioral symptoms gradually impair daily activities, such as managing finances, completing household tasks, and maintaining hygiene. The disease is characterized by extracellular amyloid-β (Aβ) plaques, intracellular neurofibrillary tangles of hyperphosphorylated tau, synaptic loss, and neuroinflammation from chronic immune activation. Incidence increases significantly after age 65. By 2025, more than 7 million Americans aged 65 and older will be affected, with numbers expected to double by 2050. Women are disproportionately impacted, primarily due to hormonal, genetic, and longevity factors. The cost of care in the U.S. is projected to reach $412 billion in 2025, posing a substantial social and economic burden. Despite extensive research, no curative or definitively disease-modifying therapies are available (Fig. 1). Recent failures of CNS-targeted treatments, including anti-amyloid monoclonal antibodies, underscore the need to explore pathogenic factors beyond the brain [1–4].

Key milestones include the identification of microbial taxa associated with AD in human cohorts (2020), the development of animal models demonstrating that fecal microbiota transplantation (FMT) can reverse AD-like pathology (2021), the initiation of clinical trials using probiotics for mild cognitive impairment (MCI) (2022), the discovery of microbial amyloids as primers for cerebral amyloid aggregation (2023), the validation of novel biomarkers (e.g., indole-3-propionate) in AD cohorts (2024), and early-phase FMT trials reporting cognitive improvements (2025). Over the past five years, the pace of discovery has accelerated, progressing from the identification of specific microbial taxa in AD cohorts (2020) to early-phase FMT trials reporting cognitive improvements [5–10].

The limited effects of CNS therapies mean that peripheral factors, such as the gut microbiome, may one day determine neuroinflammation and AD pathology [11]. The Gut-Brain Axis Program has recently launched a white paper that provides more information about the gut-brain axis. Researchers discovered that gut microbiota, a collection of microorganisms that affect brain function and behavior, play a central role in this system [12]. New studies suggest that gut microbiota dysbiosis contributes to AD pathogenesis by increasing systemic inflammation, impairing intestinal barrier function, and enhancing amyloid and tau pathology [13]—ongoing debates center on whether microbiota alterations causally drive or merely reflect neurodegeneration [14].

Most previous reviews of the gut-brain axis in AD only describe microbiome associations or lack mechanistic detail [15, 16]. This review advances the field by providing three unique contributions that distinguish it from prior work.

First, mechanistic molecular resolution: We systematically dissect how specific gut-derived metabolites—short-chain fatty acids (SCFAs), tryptophan derivatives (kynurenine, indoles), bile acids, trimethylamine N-oxide (TMAO), and microbial amyloids—act on the cellular and molecular mechanisms underlying AD pathology. Earlier reviews treat all metabolites as a single entity and assume they are identical. This is not true; we therefore focus here on the distinct molecular pathways engaged by each class of metabolites. For example, SCFAs influence histone deacetylation and regulation of brain-derived neurotrophic factor (BDNF) levels; tryptophan metabolism shifts towards neurotoxic quinolinic acid and away from neuroprotective kynurenine; bile acids affect Farnesoid X Receptor (FXR) signaling and BBB integrity; TMAO induces NLRP3 activation; and microbial amyloids seed amyloid and induce cerebral Aβ aggregation [17–21]. This analysis suggests specific therapeutic targets.

Second, integrative multi-system framework: Rather than examining isolated pathways, we present a comprehensive framework that integrates five interconnected systems: (1) metabolic signaling (SCFAs, tryptophan, bile acids, TMAO), (2) peripheral and central immune activation (MAMPs, microglial phenotype switching, systemic inflammation), (3) vagal neural communication (afferent signaling, neuromodulation), (4) neuroendocrine regulation (HPA axis dysregulation, microbial neurotransmitter production), and (5) neurovascular integrity (BBB development and disruption). This systems-level integration reflects recent advances in understanding the gut-brain axis as a dynamic, bidirectional network where perturbations in one system cascade through others, ultimately converging on AD pathogenesis [22, 23].

Third, precision medicine and translational focus: We emphasize gut-derived metabolites as actionable biomarkers for early diagnosis and risk stratification, presenting evidence from 2023 to 2025 studies that validates plasma SCFA ratios, indole-3-propionic acid (IPA), and TMAO as predictive markers correlated with hippocampal atrophy and cognitive decline [24, 25]. Furthermore, we critically evaluate emerging precision therapeutic strategies—including strain-specific probiotics (Clostridium butyricum, Bifidobacterium longum), targeted prebiotics, FMT protocols, dietary polyphenol interventions, and pharmacological modulators (FXR agonists, IDO1 inhibitors)—with particular emphasis on personalized approaches according to individual microbiome profiles, APOE genotype, and metabolic phenotypes [26–28].

To clarify these distinctions, Table 1 summarizes key differences between our review and recent publications in this domain, specifically regarding the therapies evaluated, their methodologies, and their reported outcomes. As shown in Table 1, recent reviews have highlighted the involvement of the gut-brain axis in AD. We combine mechanistic depth (i.e., specifying receptors, signaling pathways, and cellular effects for each class of metabolites), system integration (i.e., metabolic, immune, neural, neuroendocrine, and neurovascular), and a precision medicine framework. This method connects essential mechanisms to translational strategies and a roadmap that aids targeted personalized AD prevention and treatment. Despite rapid advancements, the mechanistic basis of how gut-derived factors influence AD pathogenesis remains underdeveloped, particularly at the molecular and cellular levels. To bridge this gap, our review systematically integrates cutting-edge findings from cellular and molecular neuroscience, immunology, and microbiology, primarily published [17, 21, 22]. Nevertheless, controversies persist regarding the causality—specifically, whether alterations in the gut microbiome directly contribute to AD pathogenesis or merely correlate with disease progression—and the translational potential of microbiota-targeted interventions.

**Table 1:** Comparison of this review with recent literature on gut-brain axis in AD

Review	Year	Primary focus	Mechanistic depth	Metabolite-specific analysis	Multi-system integration	Precision therapeutics	Biomarker validation (2023–2025)	References
Current review	2025	Molecular mechanisms of gut microbiota dysbiosis and metabolites in AD pathogenesis	High: Detailed molecular pathways for each metabolite class (SCFA, tryptophan, bile acids, TMAO, bacterial amyloids) with specific receptors, signaling cascades, and cellular effects	Yes: Separate sections on SCFAs (butyrate vs. acetate duality), tryptophan (kynurenine vs. indole pathways), bile acids (FXR signaling), TMAO (NLRP3 activation), microbial amyloids (molecular mimicry)	Yes: Integrates metabolic, immune, neural, neuroendocrine, and neurovascular systems into a unified framework	Yes: Strain-specific probiotics, personalized FMT, APOE-stratified interventions, metabolite-based therapeutic targets	Yes: IPA, TMAO, SCFA ratios as validated biomarkers from recent cohorts	N/A
Seo & Holtzman	2024	Understanding the AD-associated microbiome and therapeutic strategies	Moderate: Focus on microbiome composition changes and broad therapeutic categories	Partial: General discussion of SCFAs and metabolites without detailed molecular mechanisms	Partial: Primarily microbiome-immune axis	General: Overview of probiotics and diet	Limited: Primarily pre-2023 data	[20]
Zhang et al	2024	Gut microbiota metabolites as therapeutic targets	Moderate: Focus on metabolite categories and general effects	Yes: Covers multiple metabolite classes but has less mechanistic detail on molecular pathways	Partial: Metabolic and immune pathways	Moderate: Discusses metabolite modulation strategies	Some: Includes recent metabolite studies	[21]
Park & Gao	2024	Gut-brain axis and neurodegeneration mechanisms and therapeutic potentials	Moderate: Broad coverage of mechanisms across neurodegenerative diseases	Partial: General metabolite discussion across multiple diseases	Yes: Multi-system approach, but broader scope beyond AD	Moderate: General therapeutic strategies	Limited: Broader neurodegenerative focus	[19]
Chen et al	2025	Microbiota-gut-brain axis in neurodegenerative diseases	Moderate: Comparative approach across multiple neurodegenerative conditions	Partial: Metabolite discussion not AD-specific	Yes: Multi-disease comparative framework	Moderate: Broad therapeutic overview	Some Recent biomarker discussions across diseases	[17]
Li et al	2024	Systematic review of gut microbiota changes in AD spectrum (16S rRNA)	Low: Primarily compositional analysis without mechanistic depth	No: Focus on taxonomic changes	No: Microbiome composition focus	Minimal: Not primary focus	Yes: Meta-analysis of compositional data through 2023	[25]

**Fig. 2:**
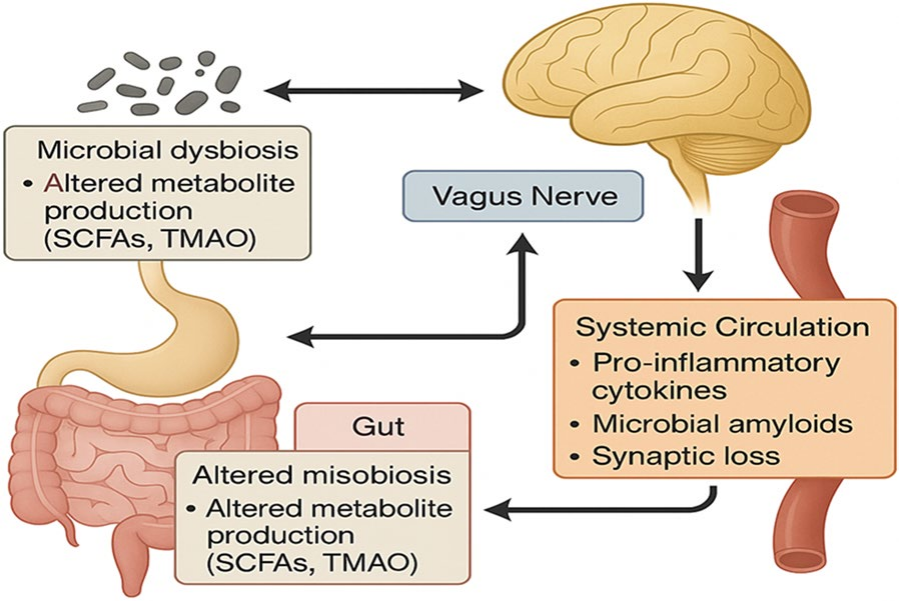
Schematic of the gut-brain axis in AD

This review aims to summarize recent findings on the gut-brain axis in AD. It aims to explain how gut bacteria and their by-products, also called metabolites, contribute to tau phosphorylation, amyloid deposition, neuroinflammation, blood–brain barrier (BBB) permeability, and other features of AD. Moreover, the review aims to identify key molecular mediators that serve as a common link between peripheral gut dysfunction and central neurodegeneration in AD. Finally, it provides a critical evaluation of novel therapeutic strategies targeting the gut-brain axis—probiotics, prebiotics, FMT, dietary interventions, and immunotherapies—to prevent or treat AD. By integrating cutting-edge findings from cellular and molecular neuroscience, immunology, and microbiology, we propose a conceptual framework elucidating how gut-derived signals shape AD pathogenesis and highlight promising avenues for future research and clinical translation. A comprehensive schematic of the gut-brain axis in AD, summarizing microbial, immune, neural, and metabolic pathways, is shown in Fig. 2.

There may be a change in the intestinal microbiota, characterized by reduced production of important microbial metabolites such as SCFAs and trimethylamine N-oxide (TMAO). These microbial products—and, in some cases, possibly bacterial amyloids—enter the systemic circulation alongside increased pro-inflammatory cytokines (right). This promotes neuroinflammation and synaptic loss in the CNS. The vagus nerve enables bidirectional signaling as changes in gut microbiota affect brain function and changes in brain function affect gut microbiota (arrow in center). Changes in the CNS affect the gut, promoting dysbiosis (bottom left). Initially, neuronal activity triggers AD pathology. SCFA; TMAO, trimethylamine N-oxide.

## The gut-brain axis: mechanisms and relevance to AD

### Components of the gut-brain axis

The Gut-Brain Axis (GBA) is a complex bidirectional communication network involving the CNS, the enteric and immune systems, and the gut microbiome. Its modulation is more prominent in the pathophysiology of AD through inflammation, amyloidogenesis, and neurodegeneration.

#### Gut microbiome: composition, diversity, and dysbiosis in AD

The gut microbiota consists of trillions of microorganisms, and the balance within the community is necessary for metabolic homeostasis and the immune system [29]. Dysbiosis is quite common in AD. It is characterized by lower diversity, lower relative abundance of Firmicutes, and an overrepresentation of Bacteroidetes and Proteobacteria [14]. Akkermansia muciniphila is a beneficial bacterium that can cause inflammation in early AD, but the mucosa is thinned in late AD [30]. On the other hand, systemic inflammation and Aβ deposition are positively related to bacteria [31]. Table 2 summarizes the main findings. This table highlights the microbial taxa identified in human cohorts and animal models, along with their correlations with amyloid-β levels and neuroinflammation.

**Table 2:** Key microbial taxa associated with Alzheimer’s disease

Microbial taxa	Study type	Key findings	References
*Akkermansia muciniphila*	Human cohort	Decreased abundance; inversely correlated with amyloid β levels	[10]
*Bacteroides*	Animal model	Increased abundance over time in 3xTg-AD mice correlates with progression of AD pathology	[9]
*Firmicutes* (phylum)	Human & animal	Decreased abundance in AD models and patients	[10]
*Bifidobacteria*	Human & animal	Decreased abundance; beneficial genus within the Actinomycota phylum	[10]
*Proteobacteria* (phylum)	Human & animal	Increased abundance associated with gut dysbiosis and neuroinflammation	[10]

*AD* Alzheimer’s disease; *3 × Tg‑AD* triple‑transgenic Alzheimer’s disease mouse model

#### Vagus nerve: direct neural pathway for gut-brain signaling

The gut and brain are directly connected via the vagus nerve, which carries microbial information—SCFAs and LPS—to the CNS. Such Vagal control, limited to transcutaneous stimulation, has shown promising effects in reducing β-amyloid burden in models and in modulating BDNF levels in AD models [32].

#### Immune activation and leaky gut

Microbial dysbiosis leads to increased gut permeability (“leaky gut”), enabling the translocation of endotoxins, such as lipopolysaccharide (LPS), into the systemic circulation. These endotoxins induce peripheral inflammation and trigger central microglial activation, thereby promoting chronic neuroinflammation—a hallmark of AD pathology [12].

#### Microbial metabolites and neuroactive compounds

Key gut metabolites produced by microbes include SCFA—butyrate, propionate, and acetate—along with tryptophan metabolites and secondary bile acids. SCFAs have been shown to play protective roles at tight junctions, as well as anti-inflammatory and neuroprotective roles, in animal models by inhibiting tau phosphorylation [33, 34]. Indole compounds derived from tryptophan can modulate microglial reactivity and serotonergic signaling and, therefore, can imply additional therapeutic targets [8]. Having established the key anatomical and molecular components of the gut-brain axis—including the microbiome composition, vagal neural pathways, immune barrier integrity, and metabolite signaling—we now turn to the empirical evidence linking gut dysbiosis directly to AD pathogenesis. The following section examines human cohort studies and preclinical models that demonstrate how alterations in these components translate into Alzheimer’s pathology, providing the translational foundation for therapeutic intervention.

### Evidence linking gut dysbiosis to AD

**Fig. 3:**
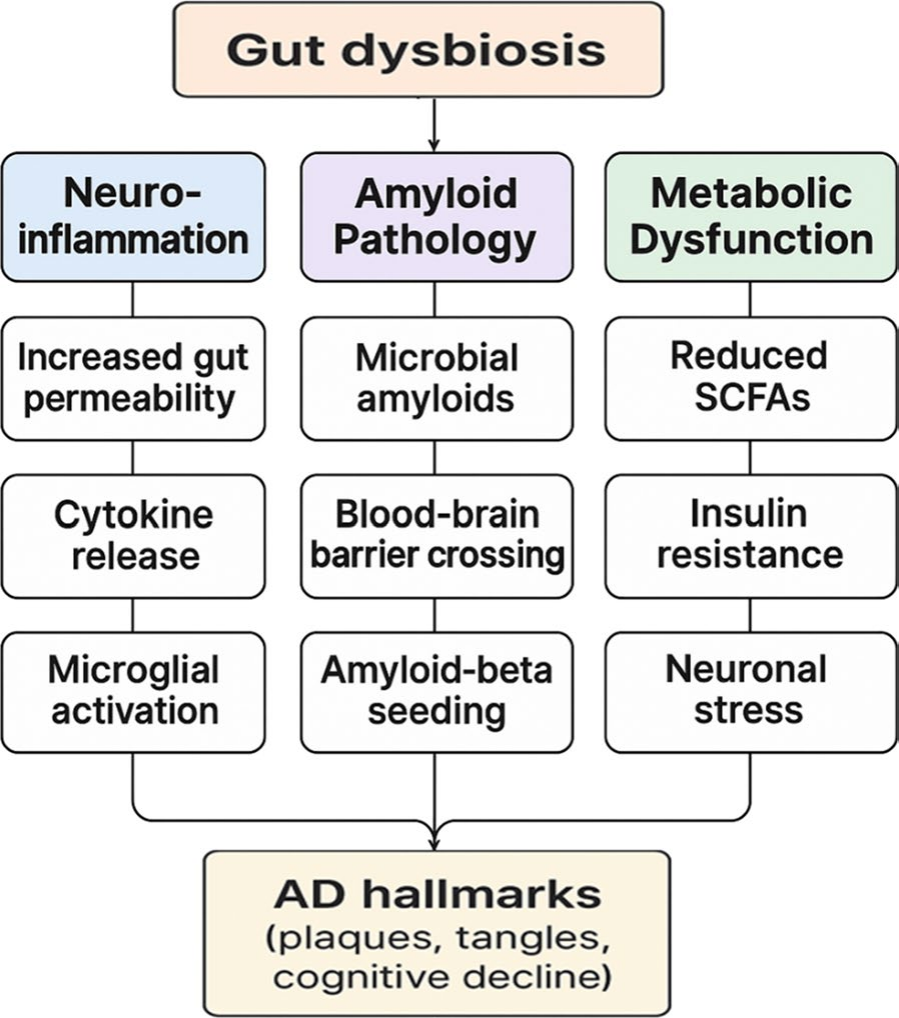
Mechanisms linking gut dysbiosis to AD pathology

An overview of these intersecting cascades is shown in Fig. 3, before we explore each in turn.

Dysbiosis of gut flora increases gut permeability so that microbial by-products and endotoxins enter the blood circulation, and consequently, systemic cytokines, microglial activation, and neuroinflammation are triggered; it forms bacterial amyloids that cross the BBB in order to initiate amyloid-beta aggregation; it decreases short-chain fatty acid production resulting in peripheral insulin resistance plus neuronal stress—these conditions combine to promote amyloid plaque formation as well as neurofibrillary tangle development alongside cognitive decline in AD. SCFA; BBB; AD, Alzheimer’s disease.

#### Human cohort studies

According to meta-analyses and clinical datasets, patients diagnosed with MCI and AD have a different gut microbiome than control subjects [35, 36]. Changes that occur early in the gut microbiome are predictive. These include a decrease in taxa that produce SCFA (such as Faecalibacterium and Roseburia) and an increase in the pro-inflammatory abundance of Escherichia and Bacteroides spp. [35]. However, differences across studies indicate the need for harmonized metagenomic approaches. However, the differences across studies highlight the need for harmonized metagenomic approaches.

#### Preclinical and translational models

AD patients ‘FMT into germ-free mice exacerbates cognitive decline, Aβ deposition, and tau aggregation [36]. In contrast, in transgenic AD models, FMT from young or healthy donors improves synaptic plasticity and reduces inflammatory markers [37]. The depletion of microbiomes by antibiotics alters tau pathology in APOE4 carriers, suggesting that host genes interact with the microbiome [38]. According to compelling evidence from human and animal studies, gut dysbiosis has been identified as a feature of Alzheimer’s disease. Creating a correlation is just the first step. A better understanding of how changes in the gut lead to damage in the central nervous system is needed to develop targeted therapies. The following subsection examines how the dysbiotic gut microbiome contributes to AD development by exploring its pathological mechanisms: neuroinflammation, amyloid-β and tau pathology, metabolic dysfunction, and oxidative stress.

### Mechanisms of gut-brain influence on AD pathology

#### Neuroinflammation

Microbial dysbiosis results in the release of systemic cytokines (IL-6, TNF-α), which subsequently activate microglia into a pro-inflammatory state [39]. Microbial metabolites and LPS cross the gut and blood–brain barriers, triggering neuroinflammatory cascades that lead to synaptic loss and neuronal death [11].

#### Amyloid and tau pathology

Bacterial amyloids (e.g., curli fibers from E. coli) may mimic or seed host Aβ aggregation via molecular mimicry, promoting cerebral amyloidogenesis [31]. Gut-derived SCFAs modulate γ-secretase activity and enhance Aβ clearance through microglial phagocytosis pathways [40]. In parallel, SCFA supplementation in tauopathy models reduces tau hyperphosphorylation and improves cognitive performance [41].

#### Insulin resistance and metabolic dysfunction

The microbiota influences glucose metabolism and insulin signaling, both of which are related to AD risk. According to SCFA, it can enhance insulin sensitivity and mitochondrial bioenergetics. In addition, it can help restore neuronal energy balance in mouse models of AD [36]. Disturbances in the gut flora contribute to peripheral insulin resistance and impose additional metabolic strain on the aging brain.

#### Oxidative stress and mitochondrial damage

A dysbiotic gut flora leads to increased reactive oxygen species and decreased antioxidant capacity in the brain. After urolithins are produced through microbial metabolism of ellagic acid, research has shown that these compounds reduce oxidative stress and improve mitochondrial turnover in AD mice [42]. Sect. “The gut-brain axis: mechanisms and relevance to AD” establishes the GBA as a bidirectional communication network fundamentally altered in Alzheimer’s disease. Evidence from human cohorts, preclinical models, and mechanistic studies indicates that gut dysbiosis is not merely a peripheral consequence of AD but an active contributor to disease pathogenesis through multiple interconnected pathways. Microbial dysbiosis drives neuroinflammation through systemic immune activation, promotes amyloid-β aggregation and tau hyperphosphorylation via bacterial amyloids and metabolite imbalances, impairs metabolic homeostasis by disrupting insulin signaling, and accelerates neuronal damage through oxidative stress and mitochondrial dysfunction [43, 44]. These insights validate the gut microbiome as a therapeutically actionable target and lay the groundwork for examining how individual gut-derived factors influence AD pathology. In Sect. “Mechanistic pathways: how the gut influences the AD brain”, these specific molecular pathways are discussed in detail, further building on the foundations established above.

## Mechanistic pathways: how the gut influences the AD brain

### Metabolic signaling pathways

Microbial metabolites from the gut act as signaling molecules, facilitating communication between gut microbes and the brain [45]. These bioactive substances can affect the brain in Alzheimer’s disease, with compounds such as SCFAs, tryptophan derivatives, and bile acids potentially playing key roles [46]. Understanding how SCFAs and other bioactive compounds function could lead to new treatments for AD [45]. Recent data suggest that the signaling pathways for these metabolites are interconnected. They appear to work together through shared targets that affect microglia, astrocytes, neurons, and cerebrovascular endothelium. By clarifying their actions through receptors—such as SCFA-induced GPR43/GPR41 activation, indole-induced Aryl Hydrocarbon Receptor (AhR) activation, bile acid-induced FXR activation, and TMAO-induced NLRP3 inflammasome activation—we can enhance the precision of therapeutic targeting of the microbiota-GBA in AD [17, 47]. To enhance understanding of the complex metabolite-mediated mechanisms linking gut dysbiosis to AD pathogenesis, Table 3 offers an integrated summary of how specific microbial metabolites converge on three critical pathophysiological domains: microglial activation and neuroinflammation, insulin signaling and metabolic dysfunction, and blood–brain barrier integrity—each representing a targetable therapeutic node in the gut-brain axis.

**Table 3:** Molecular mechanisms of gut-derived metabolites in Alzheimer’s disease pathology

Metabolite class	Key examples	Primary receptors/targets	Microglial activation & neuroinflammation	Insulin signaling & metabolic function	BBB integrity & permeability	References
SCFAs	Butyrate Propionate Acetate	GPR43 (FFAR2) GPR41 (FFAR3) GPR109A HDAC inhibition	PROTECTIVE (Butyrate): Inhibits NLRP3 inflammasome Promotes M2 microglial polarization Reduces IL-1β, TNF-α, IL-6 Upregulates TREM2 for Aβ clearance DETRIMENTAL (Acetate): Induces Microglial Neurodegenerative Phenotype( MGnD) phenotype Upregulates ApoE Impairs Aβ phagocytosis	PROTECTIVE: Enhances insulin sensitivity via AMPK activation Improves glucose metabolism Restores mitochondrial bioenergetics Reduces peripheral insulin resistance	PROTECTIVE: Upregulates claudin-5, occludin, ZO-1 Stabilizes tight junction proteins Reduces BBB permeability Enhances endothelial integrity	[48–50]
Tryptophan metabolites	Indole-3-propionic acid (IPA) Indole-3-aldehyde Indole-3-acetic acid Kynurenine Quinolinic acid	AhR NMDA receptors 5-HT receptors	PROTECTIVE (Indoles): AhR activation → NF-κB inhibition Reduces microglial reactivity Suppresses pro-inflammatory cytokines Antioxidant effects DETRIMENTAL (Quinolinic acid): NMDA receptor overactivation Excitotoxicity Oxidative stress Promotes tau phosphorylation	PROTECTIVE (IPA): Modulates insulin signaling Reduces oxidative stress Enhances neuronal energy metabolism DETRIMENTAL (Kynurenine pathway): Impairs glucose utilization Mitochondrial dysfunction	PROTECTIVE (IPA, IAA): Preserves BBB integrity Reduces endothelial inflammation Prevents tight junction disruption AhR-mediated barrier protection DETRIMENTAL (Quinolinic acid): Increases BBB permeability Oxidative endothelial damage	[51–54]
Bile acids	Deoxycholic acid (DCA) Lithocholic acid (LCA) Tauroursodeoxycholic acid (TUDCA)	Farnesoid X receptor (FXR) Takeda G-protein-coupled Receptor 5/GPBAR1 (TGR5)(GPBAR1) Pregnane X Receptor (PXR)	DETRIMENTAL (DCA, LCA): FXR-mediated astrocyte activation Microglial pro-inflammatory polarization Increased IL-1β, TNF-α PROTECTIVE (TUDCA): Reduces reactive gliosis Anti-inflammatory signaling Enhances Aβ clearance via IDE upregulation	DETRIMENTAL (DCA): Impairs insulin receptor signaling Induces peripheral insulin resistance Mitochondrial stress PROTECTIVE (TUDCA): Improves insulin sensitivity Enhances glucose metabolism ER stress reduction	DETRIMENTAL (DCA, LCA): Downregulates claudin-5, occludin Increases BBB permeability Promotes endothelial dysfunction PROTECTIVE (TUDCA): Stabilizes BBB integrity Reduces vascular inflammation Protects endothelial cells	[55–57]
Trimethylamine N-oxide (TMAO)	TMAO	NLRP3 inflammasome Protein Kinase RNA-like Endoplasmic Reticulum Kinase/Activating Transcription Factor 4 (PERK/ATF4)pathway Mitochondrial targets	DETRIMENTAL: Direct NLRP3 inflammasome activation ASC oligomerization IL-1β release from microglia Promotes M1 microglial phenotype Exacerbates astrogliosis Impairs microglial Aβ phagocytosis	DETRIMENTAL: Induces insulin resistance Impairs insulin receptor substrate-1 (IRS-1) signaling Mitochondrial dysfunction Oxidative stress in neurons Disrupts glucose homeostasis	DETRIMENTAL: Increases BBB permeability Oxidative damage to endothelial cells Disrupts tight junction integrity Promotes cerebrovascular inflammation Facilitates peripheral immune cell infiltration	[53, 58–62]
Microbial amyloids	Curli fibers (E. coli) Surfactin (B. subtilis)	TLR2/TLR4 NF-κB pathway α-synuclein cross-seeding	DETRIMENTAL: TLR2-mediated NF-κB activation Pro-inflammatory cytokine cascade Microglial priming Chronic neuroinflammation Synergistic with host amyloid aggregation	DETRIMENTAL: Indirect metabolic impairment via inflammation ER stress induction Mitochondrial membrane disruption (surfactin) Neuronal energy crisis	DETRIMENTAL: Molecular mimicry seeds cerebral Aβ aggregation Vagal transmission amplifies CNS inflammation Disrupts BBB via inflammatory signaling Facilitates amyloid infiltration	[31, 63–65]
Lipopolysaccharide (LPS)	Bacterial endotoxin (Gram-negative bacteria)	TLR4 CD14 MD-2 complexes	DETRIMENTAL: TLR4 activation on microglia MyD88-dependent inflammatory signaling Cytokine storm (IL-6, TNF-α, IL-1β) Microglial priming and chronic activation Impaired Aβ clearance	DETRIMENTAL: Induces systemic insulin resistance IRS-1 serine phosphorylation JNK/IKK pathway activation Impairs neuronal insulin signaling Metabolic endotoxemia	DETRIMENTAL: Severe BBB disruption Endothelial activation (ICAM-1, VCAM-1) Matrix metalloproteinase activation Tight junction degradation Facilitates leukocyte infiltration	[63, 66–69]

AhR, aryl hydrocarbon receptor; AMPK, AMP-activated protein kinase; Aβ, amyloid-beta; BBB, blood–brain barrier; DCA, deoxycholic acid; ER, endoplasmic reticulum; FXR, farnesoid X receptor; GPR, G-protein coupled receptor; HDAC, histone deacetylase; IAA, indole-3-acetic acid; IDE, insulin-degrading enzyme; IL, interleukin; IPA, indole-3-propionic acid; IRS-1, insulin receptor substrate-1; LPS, lipopolysaccharide; MGnD, microglial neurodegenerative phenotype; NLRP3, NOD-like receptor protein 3; SCFA; TLR, Toll-like receptor; TMAO, trimethylamine N-oxide; TNF-α, tumor necrosis factor-alpha; TUDCA, tauroursodeoxycholic acid; ZO-1, zonula occludens-1

As illustrated in Table 3, gut-derived metabolites exhibit significant mechanistic diversity and context-dependent effects on AD pathology. Notably, several key patterns emerge from this integrated analysis.

First, bidirectional metabolite effects: Within the SCFA family, butyrate demonstrates predominantly neuroprotective actions through HDAC inhibition and GPR43/109A signaling, whereas acetate can paradoxically promote neurodegenerative microglial phenotypes under specific conditions (dysbiosis, APOE4 background). Similarly, tryptophan metabolism exhibits a critical bifurcation: microbial indole derivatives (IPA, IAA) activate protective AhR signaling, while host-derived kynurenine pathway metabolites (quinolinic acid) drive neurotoxicity and tau pathology [51, 53].

Second, convergent targeting of three critical pathophysiological nodes: Despite originating from distinct microbial pathways, multiple metabolites converge on three key areas: microglial activation states (SCFAs, TMAO, LPS, bile acids), insulin signaling dysfunction (SCFAs, TMAO, bile acids, LPS), and BBB integrity (SCFAs, indoles, bile acids, TMAO, LPS). This mechanistic convergence explains how various patterns of gut dysbiosis can lead to similar AD phenotypes and suggests that therapeutic interventions targeting these nodal points may be effective across diverse patient populations [48, 49].

Third, receptor-mediated specificity enabling precision therapeutics: Each metabolite class acts through distinct receptor systems—GPR43/41/109A for SCFAs, AhR for indoles, FXR/TGR5 for bile acids, NLRP3 for TMAO, and TLR2/4 for microbial structural components. This receptor diversity enables the development of targeted pharmacological interventions (e.g., FXR agonists, AhR modulators, NLRP3 inhibitors) that can complement microbiome-based therapies, offering multimodal treatment strategies tailored to individual metabolic profiles [53, 55]. The following subsections (3.1.1–3.1.5) provide detailed mechanistic discussions of each metabolite class, building upon this integrated framework to elucidate specific molecular pathways, dose–response relationships, temporal dynamics, and translational implications for therapeutic development.

#### SCFA

SCFAs, including acetate, propionate, and butyrate, are microbial fermentation products derived from dietary fiber that play a critical neuromodulatory role in AD. In AD, gut dysbiosis alters patterns of SCFA production, typically leading to decreased butyrate-producing taxa, such as Faecalibacterium prausnitzii, and increased acetate concentrations, which correlate with β-amyloid (Aβ) aggregation and tau hyperphosphorylation [70, 71]. SCFAs cross the BBB via monocarboxylate transporters (MCTs) and modulate gene expression through epigenetic mechanisms, particularly by inhibiting histone deacetylase (HDAC). Butyrate is a potent class I and IIa HDAC inhibitor that increases neurotrophic factor (BDNF) transcription and improves synaptic plasticity and memory performance [72]. In rodent models of Alzheimer’s disease (e.g., APP/PS1 and 5xFAD), butyrate administration has been shown to reverse Aβ-induced cognitive impairment by restoring hippocampal BDNF expression and activating downstream CREB/TrkB signaling pathways [73]. However, elevated systemic SCFAs, mainly acetate, in specific-pathogen-free (SPF) mice have been associated with disease exacerbation. Acetate can induce a pro-inflammatory microglial phenotype characterized by MGnD-like transitions and upregulated ApoE, impairing Aβ clearance [74].

This context-dependent duality—in which microbial composition, host genotype (e.g., APOE4), and metabolite concentrations determine SCFA effects—underscores the need for precision microbiome therapeutics. Interventions such as targeted probiotics (Clostridium butyricum, Lactobacillus plantarum) and dietary prebiotics have been shown in both preclinical and early clinical studies to normalize SCFA levels and reduce neuroinflammation [57, 71].

#### Tryptophan metabolism

Tryptophan (Trp), the essential aromatic amino acid, is an important substrate at the Gut Microbiome (GM)–brain axis level. It is degraded via three main pathways: (1) the kynurenine pathway (KP), which leads to neuroactive and neurotoxic metabolites such as quinolinic acid (QA); (2) the serotonin/melatonin pathway, which is responsible for neuroprotection and mood regulation; and (3) the indole pathway, in which intestinal microorganisms produce immunomodulatory and antioxidant compounds such as indole-3-propionic acid (IPA) [56, 75]. A skewing of Trp metabolism toward the KP occurs in AD, where increased activity of indoleamine 2,3-dioxygenase (IDO1) converts Trp to quinolinic acid, a recognized NMDA receptor agonist and oxidant, thereby increasing tau phosphorylation and neuroinflammation [48, 76]. This imbalance is consistent with decreased serotonin levels and leads to cognitive and neuropsychiatric symptoms often associated with AD [77]. On the other hand, microbial-derived indole derivatives, including IAA, indole-3-aldehyde, and IPA, inhibit NF-κB signaling and reduce microglial activation by interacting with the AhR, thereby preventing hippocampal neurodegeneration [78]. In addition, both IPA and its analogs have been shown to inhibit Aβ fibrillization and increase neuronal antioxidant capacity [58]. Certain gut bacteria, including Bifidobacterium longum, can convert tryptophan into tryptamine, an agonist of the serotonin receptor that promotes hippocampal neurogenesis and synaptic plasticity in 5xFAD mouse models [59, 75]. Clinical and preclinical evidence increasingly suggests that therapy focusing on Trp metabolism through probiotics, Trp supplementation, or IDO1 inhibitors is a viable strategy for rebalancing the GBA and modulating neuroinflammation in early AD.

#### Bile acids and cholesterol metabolism

The gut microbiome influences cholesterol metabolism and bile acid (BA) conversion, with implications for systemic and neuroimmune responses relevant to AD. The liver produces classical bile acids, including cholic acid and chenodeoxycholic acid, which are metabolized into secondary bile acids (such as deoxycholic and lithocholic acid) via microbial 7α-dehydroxylation. These microbial metabolites can cross the BBB and further regulate brain physiology, directly and indirectly via nuclear receptor activation [61, 64].

In AD, gut dysbiosis alters bile acid pools, notably reducing Bacteroides-driven transformation, leading to elevated neurotoxic deoxycholic acid, which increases BBB permeability by downregulating tight junction proteins such as claudin-5 and occludin. Simultaneously, this BA activates astrocytes and microglia via FXR signaling, exacerbating neuroinflammation [62, 65].

Conversely, tauroursodeoxycholic acid (TUDCA), a conjugated secondary BA with neuroprotective properties, can enhance Aβ clearance by upregulating insulin-degrading enzyme (IDE) and reducing reactive gliosis in APP/PS1 mouse models [67, 68]. Recent findings also implicate FXR not only in inflammation control but also in lipid metabolism and neuronal autophagy regulation, indicating a multifaceted involvement in AD pathophysiology [69, 78].

Interventions such as FXR agonists, BA sequestrants, or microbiome engineering are under investigation as methods to rebalance the BA pool and restore BBB function. These approaches aim to normalize BA signaling cascades, thereby modifying the GBA in early and prodromal AD stages [61, 64, 68].

#### Trimethylamine N-oxide (TMAO)

Trimethylamine N-oxide (TMAO) is a microbiome product formed when trimethylamine (TMA) undergoes hepatic oxidation. TMA is made in the gut from your food (e.g., choline, carnitine, and betaine). Microbial enzymes make TMA from supplements and food. TMAO levels are significantly elevated in AD patients, and studies demonstrate that plasma TMAO concentrations greater than five μM correlate with faster hippocampal atrophy, cognitive deficits, and microglial activation in humans and in APP/PS1 mouse models [60, 79]. In APP/PS1 mice, the microbiota showed a greater abundance of TMA-producing species with age, and plasma TMAO levels increased markedly. NLRP3 inflammasome activation leads to IL-1β production by microglia and astrogliosis in the hippocampus, worsening neuroinflammation [76]. TMAO also crosses the BBB and induces oxidative stress via mitochondrial dysfunction in microglia, thereby impairing Aβ clearance [80]. Investigations conducted found that Lactobacillus plantarum administration and exercise reduced TMAO levels and restored hippocampal synaptic plasticity by altering the gut microbiota composition to fewer TMA-producing microbes [36, 81]. Importantly, TMAO has recently been identified as a biomarker of early cognitive impairment, underscoring the need for clinical risk stratification [39].

#### Microbial amyloids and neurotoxins

The microbes in the gut produce proteins and harmful substances that affect brain health. Curli fibers produced by E. coli exhibit structural homology with host β-amyloid (Aβ) and have been shown to override Aβ aggregation both in vitro and in vivo. Curli amyloids do the following: They activate TLR2-mediated NF-κB signaling, thereby activating neuroinflammatory cascades and tau phosphorylation [63, 82].

In Tg2576 AD mice, colonization with curli-producing E. coli significantly increases ileal expression of PGP9.5 + neuroendocrine cells, stimulating afferent vagal pathways that relay pro-inflammatory signals to the nucleus tractus solitarius (NTS), resulting in enhanced hippocampal Aβ burden (Tić, 2022). This gut-to-brain relay exemplifies the bottom-up inflammatory signaling that may precede central amyloidosis. Bacillus subtilis, another common spore-forming commensal, secretes surfactin (a cyclic lipopeptide). This molecule acts as a potent neurotoxin, disrupting the mitochondrial membrane potential and damaging neurons. The toxicity of surfactin is enhanced by conditions that disrupt BBB permeability, which is frequently observed in AD [83].

Fermented meals containing Bacillus subtilis or E. coli can either harm or remedy AD pathology, depending on the amyloid, metabolic context, and immune tone [82, 83]. These findings underscore the need for strain-level characterization and urge caution against the blanket use of live biotherapeutics in neurodegeneration. This gut-to-brain amyloid relay exemplifies how structural mimicry between microbial and host proteins can initiate pathological cascades, highlighting the need for strain-specific microbiome characterization in AD risk assessment [84].

Collectively, these microbial metabolites function as an integrated signaling network that converges on common molecular targets, including nuclear receptors (FXR, AhR), G-protein-coupled receptors (GPR43, GPR41, GPR109A), inflammasome complexes (NLRP3), and epigenetic regulators (HDACs). Recent systems biology approaches reveal extensive crosstalk between metabolite-activated pathways: for instance, SCFAs and bile acids synergistically regulate BBB permeability through complementary effects on tight junction proteins; tryptophan-derived indoles and SCFAs converge on microglial polarization via AhR and GPR43 co-activation; and TMAO amplifies inflammatory cascades initiated by bacterial LPS. This mechanistic integration underscores the rationale for multi-targeted therapeutic approaches—including synbiotic formulations combining SCFA-producing and indole-producing strains, dietary interventions that simultaneously modulate multiple metabolite pathways, and precision medicine strategies that account for individual variation in metabolite production, receptor expression, and host genotype (particularly APOE4 status). The context-dependent effects of these metabolites—protective versus pathogenic depending on concentration, timing, microbial community structure, and host factors—emphasize the need for personalized microbiome-based diagnostics and therapeutics in AD management. While gut-derived metabolites represent critical biochemical mediators of gut-brain communication in AD, these molecular signals do not act in isolation. Instead, they converge on and are amplified through systemic and central immune activation—the second major mechanistic axis linking the gut to the Alzheimer’s brain. The following section examines how microbial products trigger peripheral inflammatory cascades, reprogram microglial phenotypes, and perpetuate chronic neuroinflammation, thereby accelerating disease progression.

### Immune system mediation

#### Microbe-associated molecular patterns (MAMPs) and neuroinflammation

MAMPs, such as lipopolysaccharide and polysaccharide A, play a central role in the development of neuroinflammation via the gut-brain-immune axis. In AD, increased systemic concentrations of LPS from Gram-negative bacteria overcome the leaky gut-BBB and activate TLR4 on microglia and perivascular macrophages, leading to pro-inflammatory signaling and overproduction of TNF-α and IL-6 [85, 86].

This LPS-TLR4 axis causes microglial priming, synaptic pruning, and accelerated Aβ deposition in transgenic AD models such as 5xFAD. Mice aged 5xFAD exhibit increased gut permeability, allowing leakage of LPS that activates glia in the hippocampus [87].

In contrast, polysaccharide A (PSA), which is produced by Bacteroides fragilis, activates Tregs, particularly IL-10–secreting Foxp3⁺-Tregs. These cells cross the BBB and inhibit the activity of reactive astrocytes and the release of pro-inflammatory cytokines in the CNS. Treatment with PSA restores the immune system’s balance in the aged AD brain, making it a potential next-generation postbiotic therapy. The difference between LPS, which triggers inflammation, and PSA, which prevents inflammation, shows how gut-derived MAMPs can affect the immune response in both directions. This means that the effect of microbes in AD is reversible. However, the microbes must be administered precisely for modulation of AD to occur [86].

#### Microglia activation states in response to gut signals

SCFAs are revealed as crucial mediators that stimulate microglial activation in AD and mediate both protective and deleterious effects [47]. Microglia, the brain’s resident immune cells, can shift between anti-inflammatory (homeostatic/M2-like) and pro-inflammatory (neurodegenerative/MGnD) phenotypes in response to microbial metabolites [88]. Microglia, the brain’s immune cells, can switch between anti-inflammatory (homeostatic/M2-like) and pro-inflammatory phenotypes.

In particular, butyrate induces microglial homeostasis through mechanisms independent of direct GPR43 receptor activation, as primary microglia do not express FFAR2 (GPR43) [47, 89]. Instead, SCFAs modulate microglial function through histone deacetylase (HDAC) inhibition and monocarboxylate transporter-mediated uptake, subsequently inhibiting the NLRP3 inflammasome by decreasing ASC oligomerization and IL-1β release [41, 89]. Butyrate supplementation restores microglial phagocytic capacity in germ-free APP/PS1 mice, associated with upregulation of TREM2 and CD33, two important receptors involved in Aβ clearance and AD risk [63].

Conversely, acetate—another SCFA—has been shown to promote a neurodegenerative microglial (MGnD) transcriptional profile, increasing Apo E expression and promoting a disease-associated signature that impairs Aβ phagocytosis and accelerates plaque accumulation [41]. This emphasizes the metabolically specific and context-dependent nature of gut-derived SCFAs’ effects on CNS immunity. Modulating the SCFA–microglial axis through diet, targeted prebiotics, or SCFA analogs represents a promising strategy to rebalance immune tone in early-stage AD.

#### Systemic inflammatory signaling

The gut microbiome contributes to and influences AD through gut-brain exchanges and the systemic inflammatory response. Serum amyloid A (SAA) is one of this axis’s most important molecular mediators. It is an acute-phase reactant that increases intestinal dysbiosis. SAA binds to the receptor for advanced glycation end products on cerebral endothelial cells, allowing the influx of β-amyloid (Aβ) into the brain parenchyma (i.e., brain cells) and activating astrocytic and microglial cytokine cascades [90].

At the same time, gut immune cells, especially Th17 cells, are influenced by specific microbial groups, such as segmented filamentous bacteria (SFB). Interleukin-17A (IL-17A) is a cytokine that enters the CNS and stimulates C1q expression at synapses to initiate complement-mediated synaptic pruning, which is pathologically accelerated in early AD [91, 92]. C1q activity is too much. When this happens, it leads to accelerated synapse loss. This is particularly true in the hippocampus and cortex [92].

Recent studies demonstrate that manipulating Th17 expansion via diet, probiotics, or RORγt inhibition can reduce IL-17A levels and preserve synaptic integrity, suggesting that peripheral immune control may offer upstream leverage over central pathology [90, 93]. Beyond metabolite diffusion and systemic immune signaling, the gut communicates with the brain through a third, anatomically discrete pathway: direct neural transmission via the vagus nerve. This bidirectional neural highway enables rapid, real-time communication between gut microbiota and central nervous system structures, offering unique therapeutic opportunities for neuromodulation. We now examine the vagal mechanisms through which microbial signals are transduced into neurophysiological responses relevant to AD pathogenesis. While vagal neural signaling provides rapid, direct communication between gut and brain, the microbiota also influences central nervous system function through hormonal and neurotransmitter pathways that operate on slower timescales but exert profound regulatory control. The neuroendocrine system, particularly through modulation of the hypothalamic–pituitary–adrenal axis and microbial neurotransmitter production, represents a fourth critical mechanism linking gut dysbiosis to AD pathology.

### Neural signaling through the vagus nerve

#### Afferent vagal communication from gut-to-brain

The vagus nerve is a primary bidirectional communication pathway between the gut microbiota and the brain. It transmits microbial signals that influence the neuroimmune and neuroendocrine pathways involved in AD [32].

Curli amyloids produced by Escherichia coli are involved in the crucial interaction. Toll-like receptor 2 (TLR2) on enteroendocrine and immune cells of the intestinal epithelium is activated by these extracellular fibers. Curli, which activates vagal afferents, sends signals to the NTS and locus coeruleus that alter the release of noradrenergic (NE) in the hippocampus and increase the expression of IGF-1 [93, 94]. According to the APP/PS1 mouse model (80, 81), both NE and IGF-1 can promote Aβ clearance. Consequently, these agents can either enhance microglial activation or upregulate IDE [95, 96].

The composition of the gut microbiota also influences the overall degree of vagal tone and the intensity of afferent signaling. A lack of the right bacteria impairs vagal A system function, whereas the appropriate bacterium, Lactobacillus rhamnosus, restores system responses and improves memory in animal brains [97].

These results emphasize that afferent vagal activation by microbial products may serve as a neuroprotective feedback loop that counteracts AD pathology by modulating neurotransmitters and growth factors.

#### Neuromodulatory effects relevant to AD

Vagus nerve stimulation (VNS) has developed into a neuromodulatory treatment modality with anti-inflammatory potential in AD, which it exerts essentially by engaging the α7 nicotinic acetylcholine receptor (α7nAChR) on microglia and astrocytes. Activation of α7nAChRs results in inhibition of pro‐inflammatory cytokines, notably IL‐1β and TNF‐α, and a return toward microglia homeostasis [98, 99]. Transcutaneous VNS significantly improved spatial memory performance in 5xFAD mice and diminished hippocampal plaque burden. This was concomitant with enhanced α7nAChR expression in microglia and activation of anti-inflammatory signaling pathways, such as the JAK2/STAT3 pathway [32]. Electrophysiological studies demonstrate that α7nAChR activation also modulates synaptic plasticity, particularly in the hippocampus, a region critically affected in AD [100].

Pharmacologic enhancement of α7nAChRs or targeted VNS results in neuroprotective effects, including reductions in oxidative stress, restoration of long-term potentiation (LTP), and improved cholinergic transmission in AD models [101]. The cholinergic anti-inflammatory reflex, centrally mediated via the vagus nerve, offers a bidirectional therapeutic target with both peripheral and central anti-inflammatory impact [102].

#### Bacterial signaling via vagal afferents

Recent findings reveal that Lactobacillus rhamnosus JB-1 communicates with the brain via extracellular vesicles (EVs) that carry small RNAs and miRNAs, which engage host neuroimmune networks. These EVs can modulate vagal nerve activity by acting on enteroendocrine cells and primary afferent neurons, thereby transmitting signals to the brainstem and hippocampus [103, 104].

In preclinical studies, L. rhamnosus JB-1 has been shown to influence central neurotransmitter systems and reduce neuroinflammatory markers in the brain via vagus nerve–dependent mechanisms, although direct validation in AD models is still limited. One proposed mechanism involves miR-155–containing EVs, which may downregulate pro-inflammatory genes, such as BACE1, a β-secretase involved in Aβ production [105].

While specific confirmation in APP/PS1 or 5xFAD models is pending, mechanistic extrapolations from major depressive disorder (MDD) and stress-related neuroinflammation studies strongly support a role for microbiota-derived miRNAs and EVs in the modulation of CNS targets by the vagus [106, 107].

### Neuroendocrine system communications

#### HPA axis dysregulation

The GBA is closely linked to the HPA axis. HPA refers to the hypothalamic–pituitary–adrenal axis. It is a neuroendocrine structure, according to some researchers. HPA observes personality types. HPA is associated with AD. In APP/PS1 mouse models, gut microbiota depletion increases circulating corticosterone levels, the murine analog of cortisol. This leads to increased transcription of β-secretase 1 (BACE1), mediated by glucocorticoid receptor (GR) binding to its promoter. This ultimately leads to accelerated Aβ42 deposition [108, 109].

Chronic activation of the HPA axis leads to phosphorylation of the GR in the hippocampus, impairing feedback inhibition, promoting persistent neuroinflammation, and decreasing synaptic plasticity [34]. Notably, probiotic interventions—particularly using Bifidobacterium infantis and Bifidobacterium longum—have shown promise in normalizing HPA axis activity, restoring hippocampal GR function, and reducing Aβ burden in transgenic models [110, 111]. These findings suggest that targeted microbiome modulation may serve as a therapeutic avenue to recalibrate neuroendocrine stress pathways in early-stage AD and attenuate downstream amyloidogenic signaling.

#### Microbial neurotransmitter production

The gut microbiota influences the CNS by producing and modulating the neurotransmitters GABA and serotonin [103].

Lactobacillus spp. Including L. GABA-producing Lactobacillus strains, such as Rhamnosus, has been shown to help attenuate the cortical hyperexcitability seen in early AD and MCI. Research on GABA in mouse models of AD has shown that GABA inhibits glutamatergic excitotoxicity and restores neuronal network stability [112, 113]. In addition, tryptophan deficiency is caused by Candida albicans overgrowth. As a result, tryptophan is diverted from serotonin production. At the same time, this diversion leads to kynurenine pathways. The kynurenine pathways, therefore, produce neurotoxins that exacerbate depression-like behaviors and cognitive impairment [114].

Furthermore, gut-derived serotonin has been shown to regulate hippocampal neurogenesis and plasticity, and interventions that restore microbial tryptophan metabolism—such as administration of Bifidobacteria or polyphenol-rich diets—have reversed mood and memory deficits in experimental models [115, 116]. The metabolic, immune, neural, and neuroendocrine pathways we examined converge on a critical structure: the BBB. The BBB maintains brain homeostasis. Its integrity determines whether peripheral signals—beneficial or pathogenic—can reach the central nervous system. Next, we will explore how gut dysbiosis disrupts this neurovascular interface. This disruption creates a pathway for peripheral inflammation and microbial products to enter the brain parenchyma and contribute to Alzheimer’s disease pathology.

### BBB integrity

#### Microbiota regulation of BBB development and function

The BBB and gut microbial signals are vital in health and disease. Mice raised in a germ-free environment do not develop a BBB. They have low claudin-5 and occludin, which are tight junction proteins. Giving them SCFA helps reverse this situation. SCFA supplementation can be achieved by adding an important fatty acid, butyrate [117, 118].

Butyrate enhances BBB integrity by activating the AMPK pathway, stabilizing TJ proteins, and inhibiting MLCK-mediated cytoskeletal destabilization. In models of AD, activation of AMPK decreased BBB permeability and inflammation-induced leakage, making it a potential therapy to prevent early-onset Aβ extravasation [119, 120]. These mechanisms directly affect endothelial cell gene expression through epigenetic rearrangements, while also indirectly reducing peripheral inflammation and maintaining the integrity of the neurovascular unit. Microbial metabolites induce the release of gut-brain signaling molecules that influence neuronal circuits in the brain and/or modulate the BBB workings.

#### Mechanisms of BBB disruption in dysbiosis

Gut dysbiosis leads to BBB damage in AD by increasing systemic inflammation and enhancing the generation of microbial-derived toxins, including LPS [121]. LPS induces toll-like receptors in brain micro-vascular endothelium, which promotes the production of matrix metalloproteinase-9 (MMP-9). Type IV collagen and tight junction proteins (occludin and claudin-5) are enzymatically degraded by MMP-9, increasing BBB permeability and promoting Aβ40 extravasation into the brain parenchyma [122].

Enhanced MMP-9 activity is also associated with decreased expression of zonula occludens-1 (ZO-1), a critical scaffolding protein necessary for the maintenance of TJ. This impairment results in the infiltration of neurotoxic proteins and peripheral cytokines into the brain and could exacerbate amyloidogenic and tau-related pathology [123]. By contrast, the gut bacterium A. muciniphila demonstrated neuroprotective effects on the BBB, including in APOE4 transgenic models. This defense is, at least in part, due to the released peptide P9, which induces ZO-1 preservation and stabilizes endothelial junctions. Therein, modulation of gut-derived peptides represents a potential therapeutic approach to reconstitute neurovascular integrity in dysbiosis-driven AD [124, 125]. Sect. “Mechanistic pathways: how the gut influences the AD brain” has dissected the multifaceted mechanistic architecture through which the gut microbiome influences Alzheimer’s disease pathogenesis, revealing an integrated systems-level framework rather than isolated pathways. Five interconnected mechanisms collectively orchestrate gut-brain communication: (1) metabolic signaling through SCFAs, tryptophan derivatives, bile acids, TMAO, and bacterial amyloids that directly modulate neuronal function, epigenetic regulation, and amyloid aggregation; (2) immune system activation through MAMPs and microglial polarization that drives chronic neuroinflammation; (3) vagal neural transmission providing rapid bidirectional gut-brain signaling and neuromodulatory control; (4) neuroendocrine regulation via HPA axis dysregulation and microbial neurotransmitter production affecting stress responses and synaptic plasticity; and (5) BBB compromise enabling peripheral inflammatory mediators to infiltrate the CNS [40, 43].

## Clinical and translational evidence

**Fig. 4:**
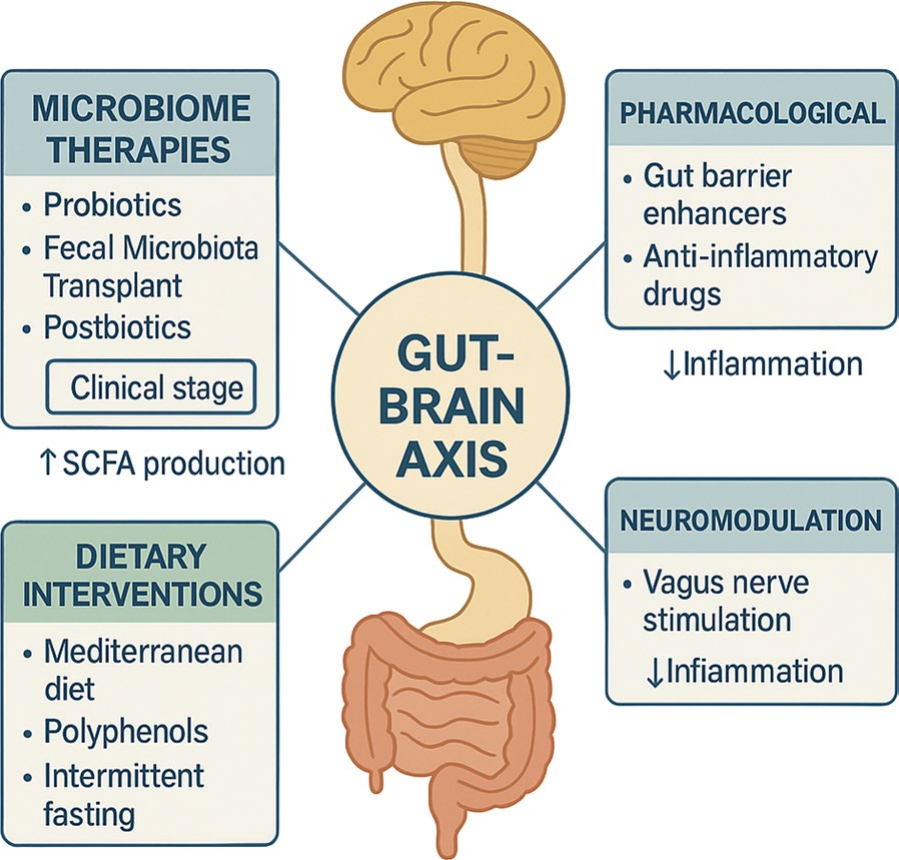
Therapeutic strategies targeting the GBA

As summarized in Fig. 4, a multi-pronged toolkit is emerging to rebalance dysbiotic gut-brain communication in AD before we review each modality in depth.

This figure shows four major intervention categories to modulate the GBA for possible therapeutic application in AD. Microbiome interventions, including probiotics, FMT, and postbiotics, aim to enhance SCFA production and are in the early stages of clinical development. Pharmacological interventions comprise gut barrier stabilizers and anti-inflammatory agents licensed for clinical application and aim to reduce systemic inflammation. Dietary regimens include a Mediterranean diet, polyphenol-rich foods, and intermittent fasting, which increase SCFA levels and antioxidative activity. Neuromodulation is VNS preclinical development, which may target reduced neuron signaling and inflammation. All interventions target the GBA; hence, they play a central role in modulating neuroinflammation, leading to cognitive outcomes in AD.

### Observational studies

Recent cross-sectional and longitudinal studies consistently show gut microbiota dysbiosis in AD, even during its preclinical stages. For example, a case–control study in Uganda found that patients with AD exhibited significantly lower gut microbial diversity and higher abundance of Proteobacteria and Bacteroides, correlating with MMSE scores and ApoE genotype [126].

In a Chinese cohort of 100 MCI and AD patients, 16S rRNA sequencing revealed that Blautia and Escherichia–Shigella were enriched, while beneficial taxa such as Faecalibacterium were depleted, changes that aligned with neuroinflammatory and cognitive markers [127]. Meanwhile, Prevotella abundance was reduced in several Western cohorts, though findings vary by geography and diet, underscoring the importance of regional gut microbiome baselines [128].

Another recent prospective study by Climacosa et al. (2024) highlighted that Parabacteroides distasonis and circulating SCFA levels were associated with hippocampal volume and early Aβ deposition in at-risk populations [129]. These findings support the use of gut microbial profiling as a non-invasive early biomarker for AD risk stratification.

In Table 4, we summarize the clinical trials targeting the GBA in AD. These trials highlight emerging microbiome-based interventions and their potential to modify AD progression. While observational studies have established robust correlations between gut microbiome alterations and AD progression in human cohorts, demonstrating causality and therapeutic potential requires interventional evidence. The following section examines clinical trials that have sought to modulate the GBA using various therapeutic modalities, providing critical translational data on feasibility, safety, and preliminary efficacy signals for microbiome-targeted AD interventions.

**Table 4:** Clinical trials targeting the GBA in AD

Intervention type	Study population	Primary outcome	Key results	Trial status	References
FMT	MCI and AD patients	Cognitive scores (MoCA, ADAS-Cog), Microbiome diversity	Improved or maintained cognitive function in MCI; no worsening in severe AD; altered gut microbiota and serum metabolites; no adverse effects reported	Completed	[130]
Probiotic supplementation	AD and MCI patients	Cognitive function, inflammation markers	Some studies report cognitive improvement and reduced inflammation; evidence is still preliminary	Ongoing/Recruiting	[20, 131]
Mediterranean diet	MCI and at-risk elderly	Cognitive scores, gut microbiome diversity	Associated with improved cognition and beneficial microbiome changes; clinical trial data emerging	Ongoing	[20, 131]
Prebiotic supplementation	AD or MCI patients	Cognitive function, gut microbiota composition	Early trials suggest modulation of microbiota and potential cognitive benefits; more data needed	Recruiting	[20]
Synbiotic (Probiotic + Prebiotic)	MCI patients	Cognitive performance, inflammatory markers	Synbiotic supplementation improved cognitive scores and reduced markers of inflammation compared to placebo	Completed	[132]
Polyphenol-rich dietary intervention	Early AD or MCI patients	Cognitive function, gut microbiota diversity	Polyphenol intervention improved memory scores and increased the abundance of beneficial gut bacteria	Ongoing	[133]

FMT: Fecal Microbiota Transplantation; MCI: Mild Cognitive Impairment; AD: Alzheimer’s disease; MoCA: Montreal Cognitive Assessment; ADAS‑Cog: Alzheimer’s Disease Assessment Scale–Cognitive Subscale

This table highlights emerging microbiome-based interventions with potential to modify AD progression via the GBA, though larger, well-powered trials are needed to confirm efficacy and safety.

### Interventional studies

Interventions targeting the gut microbiota in AD have progressed from probiotics and prebiotics to more experimental strategies, such as FMT and time-restricted eating. A 2025 umbrella review of RCTs found that multi-strain probiotic supplementation improved global cognition and memory in AD and MCI, though heterogeneity remained high [134].

FMT studies remain in early phases but are gaining traction. A pilot trial in China demonstrated that FMT from young donors into MCI patients improved gut barrier integrity and decreased plasma LPS and IL-6 levels [135]. Meanwhile, Dong et al. (2025) emphasized that probiotics also boost BDNF and antioxidant defenses, adding a mechanistic rationale for microbiome-based therapeutics [36].

Additionally, Rentz et al. (2024) showed that a multimodal lifestyle intervention (diet, exercise, mindfulness) altered microbial diversity and decreased plasma Aβ and tau markers over 24 months, showing promise as a non-invasive AD-modifying strategy [136]. In Table 5, we present gut-derived metabolites that serve as potential biomarkers for AD. These metabolites are linked to neuroinflammation and other pathological processes in AD. The mixed results from interventional studies highlight a critical challenge: without validated biomarkers of GBA dysfunction, we cannot effectively stratify patients, optimize intervention timing, or monitor therapeutic responses. Developing and validating gut-derived biomarkers for Alzheimer’s disease risk, diagnosis, and treatment response is essential for bridging the gap between mechanistic understanding and clinical translation.

**Table 5:** Gut-derived metabolites as potential AD biomarkers

Metabolite	Source (Microbial taxa/process)	Effect on AD pathology	Detection method	Clinical relevance	References
SCFAs	Produced by fermentation of dietary fiber by *Clostridiaceae*, *Faecalibacterium*	Modulate neuroinflammation, maintain BBB integrity, and regulate microglial activation	Blood, CSF, Feces	Diagnostic, Prognostic	[119, 137]
Trimethylamine N-oxide (TMAO)	Gut microbial metabolism of choline and L-carnitine by *Proteobacteria* and others	Elevated in AD; promotes neuroinflammation and amyloid pathology	Blood	Diagnostic	[18, 137]
Bile Acids (e.g., DCA, GDCA)	Microbial conversion of primary bile acids by *Bacteroidetes* and others	Altered BA profiles linked to neurodegeneration and mitochondrial dysfunction	Blood	Diagnostic, Prognostic	[137, 138]
Indole-3-Propionate	Produced by *Clostridium* species via tryptophan metabolism	Neuroprotective effects: modulates the BBB and reduces oxidative stress	Blood, CSF	Prognostic	[119, 139]
Lipopolysaccharide (LPS)	Outer membrane component of Gram-negative bacteria (e.g., *Bacteroidetes*)	Triggers neuroinflammation, BBB disruption, and microglial activation	Blood, CSF	Diagnostic	[18, 119, 137]
Sphingolipids	Microbial and host lipid metabolism	Regulate amyloid β metabolism and Tau phosphorylation	Blood, CSF	Prognostic	[119, 140]
γ-Aminobutyric Acid (GABA)	Produced by *Lactobacillus*, *Bifidobacterium*	Modulates neuroinflammation and neuronal excitability	Blood, CSF	Diagnostic	[119, 141]

AD: Alzheimer’s disease; SCFA: Short-chain fatty acids; BBB: Blood–brain barrier; CSF: Cerebrospinal fluid; TMAO: Trimethylamine N‑oxide; DCA: Deoxycholic acid; GDCA: Glycodeoxycholic acid; LPS: Lipopolysaccharide; GABA: γ‑Aminobutyric acid

This table highlights key microbial metabolites that influence AD pathogenesis by modulating neuroinflammation, amyloid and tau pathology, BBB integrity, and oxidative stress, and serve as promising biomarkers for diagnosis and prognosis.

### Biomarkers of GBA dysfunction

The field is rapidly advancing in identifying reliable biomarkers that link changes in the gut microbiome to the development of CNS diseases. Notably, the gut metabolite TMAO has been found at higher levels in blood plasma, correlating with hippocampal shrinkage and increased amyloid-beta plaques, hallmarks of AD [142]. A study using Mendelian randomization by Ning and colleagues in 2024 provided evidence for a causal role of TMAO in Alzheimer’s, also linking specific gut bacteria to reduced levels of a neuroprotective protein (BDNF) and increased BBB permeability [143]. Promising new biomarkers under investigation include lower levels of the neuroprotective microbial metabolite indole-3-propionate in blood. These PET scans can visualize BBB breakdown by targeting ZO-1 proteins, and circulating vesicles and small RNA molecules released by microbes, as reported by Drago et al. in 2025 [84]. To personalize early diagnosis of Alzheimer’s, large-scale projects like MEMORI-AD (2023) are integrating comprehensive analyses of gut microbial DNA (using 16S and shotgun metagenomics), blood metabolites, and advanced brain imaging techniques [144]. The clinical and translational evidence in Sect. “Clinical and translational evidence” supports the GBA as a relevant therapeutic pathway in human Alzheimer’s disease. However, it also highlights critical gaps that must be addressed for clinical implementation. Observational studies consistently show alterations in the gut microbiome of AD patients, including decreased microbial diversity, reduced SCFA-producing taxa, and elevated pro-inflammatory species. These changes correlate with disease severity and cognitive decline [23]. Early-phase interventional trials using probiotics, prebiotics, and dietary modifications demonstrate promising safety profiles and modest cognitive benefits in populations with MCI. However, effect sizes vary, and study heterogeneity limits the ability to draw definitive conclusions [44]. The emergence of validated gut-derived biomarkers—particularly plasma SCFA ratios, indole-3-propionic acid, and TMAO—offers diagnostic and prognostic utility, but standardization and longitudinal validation across diverse populations remain required. These findings support cautious optimism while emphasizing the necessity for larger, well-controlled trials with standardized protocols, validated biomarker endpoints, and personalized intervention strategies based on individual microbiome profiles and host genetics. Sect. “Potential therapeutic targets” will examine the specific therapeutic modalities under development to target the GBA and discuss the precision medicine frameworks needed to optimize their clinical application.

## Potential therapeutic targets

### Microbiome-targeted therapies

Different microbiome-targeted interventions, including probiotics, FMT, synbiotics, and pharmacological approaches, exhibit varying efficacy in modifying the progression of Alzheimer’s disease through gut-brain signaling, as summarized in Table 5.

#### Probiotics and prebiotics

Probiotic strains such as Lactobacillus acidophilus, L. casei, Bifidobacterium bifidum, and L. fermentum have neuroprotective properties in AD models and clinical populations. MMSE scores improved in a 12 week RCT with a multi-strain formulation, and IL-6 and MDA levels were reduced in Alzheimer’s patients [120]. Prebiotics support the establishment of these probiotics, while synbiotics enhance SCFA production and gut-brain communication. In 3xTg-AD mice, females had a greater reduction in microglial activation and better neuron preservation than males after probiotic treatment [112].

#### FMT

FMT aims to restore eubiotic gut microbial communities. A pilot clinical trial in China showed FMT improved intestinal permeability and reduced plasma LPS in MCI subjects [135]. Despite early promise, standardization of donor selection, safety assurance, and long-term efficacy data remain significant hurdles. Recent reviews emphasize the need for personalized FMT protocols, accounting for host-microbiome compatibility [135].

#### Postbiotics

Postbiotics—bioactive microbial products including SCFAs, urolithins, and indole derivatives—offer precise therapeutic benefits without live bacteria. Butyrate enhances tight junction protein expression and restores BBB function, while urolithin A supports mitochondrial function and autophagy in AD models [135]. Engineered delivery systems and synthetic biology now allow in situ biosynthesis of these metabolites in targeted brain regions (Table 6).

**Table 6:** Comparison of therapeutic approaches targeting the GBA

Approach	Mechanism of action	Stage of development	Advantages	Limitations/challenges	References
Probiotics	Modulate gut microbiota composition; reduce neuroinflammation; enhance gut barrier and immune function	Clinical Phase II/III	Safe, non-invasive; may improve cognition and inflammation	Strain-specific effects; variability in patient response; limited large-scale trials	[120, 145]
FMT	Restore healthy gut microbiota diversity; reduce systemic and neuroinflammation	Early Clinical /Preclinical	Potential for broad microbiome restoration	Safety concerns; donor variability; regulatory hurdles; small sample sizes in AD trials	[145, 146]
Dietary Interventions (e.g., Mediterranean diet, polyphenols)	Modulate gut microbiota and metabolites; reduce oxidative stress and inflammation; improve metabolic health	Clinical Phase II/III	Widely accessible; multiple health benefits	Long-term adherence challenges; heterogeneous effects; need for controlled trials	[147, 148]
Pharmacological Approaches (e.g., prebiotics, synbiotics)	Promote growth of beneficial microbes; modulate gut-derived metabolites; reduce neuroinflammation	Clinical Phase II	Targeted modulation of microbiome; potential synergy with probiotics	Limited data on optimal formulations and dosing; side effects unknown	[111, 148]
VNS	Modulates gut-brain neural communication, reduces neuroinflammation, and enhances cognitive function	Clinical Phase II/III	Direct neuromodulation: promising cognitive benefits	Invasive procedure; surgical risks; device costs; limited AD-specific data	[149, 150]
Neuromodulation (e.g., transcranial magnetic stimulation)	Modulates gut-brain neural communication; reduces neuroinflammation; enhances cognitive function	Clinical Phase II/III	Direct neuromodulation: promising cognitive benefits	Invasive procedure; surgical risks; device costs; limited AD-specific data	[151]

AD: Alzheimer’s disease; FMT: Fecal microbiota transplantation; Phase II/III: Phase II and Phase III clinical trials; Preclinical: Studies in animal models or in vitro; VNS: Vagus nerve stimulation; TMS: Transcranial magnetic stimulation

#### Dietary interventions

The Mediterranean and MIND diets are associated with increased SCFA-producing bacteria (Roseburia, Faecalibacterium) and reduced Proteobacteria. These diets are linked to slower cognitive decline and reduced AD incidence [147]. Intermittent fasting enhances neuronal resilience by promoting microbial shifts and reducing oxidative stress. Polyphenols in berries and teas upregulate indoleamine 2,3-dioxygenase inhibitors and promote the production of neuroprotective indoles [152].

#### Pharmacological approaches

Microbiome-modulating drugs are emerging. Metformin, by activating AMPK, improves insulin signaling and rebalances gut flora, reducing Aβ and tau pathology [153]. Antibiotics have been trialed but may disrupt commensal taxa. Tight junction stabilizers and gut-derived cytokine inhibitors are in development [154].

#### Neuromodulation

Non-invasive VNS enhances cholinergic anti-inflammatory pathways, elevating norepinephrine and reducing tau phosphorylation. Preclinical models show VNS improves hippocampal synaptic plasticity and reduces microglial activation, making it a candidate for symptomatic relief [155, 156].

#### Precision medicine approaches

Machine learning-based algorithms now predict microbial response patterns, enabling personalized probiotic design and dietary regimens. Integration of host genomics, microbiomics, and plasma metabolomics allows identification of subgroups for tailored therapy. Postbiotic cocktails and targeted synbiotics are being trialed as part of individualized plans [157–159]. Sect. “Potential therapeutic targets” has outlined a comprehensive therapeutic portfolio targeting the GBA in Alzheimer’s disease, ranging from direct microbiome modulation to host-directed interventions and integrated precision medicine approaches. The evidence supports a multi-modal strategy rather than reliance on single interventions: microbiome-targeted therapies (probiotics, prebiotics, FMT, postbiotics) show promise in restoring beneficial microbial taxa and metabolite production; dietary interventions—particularly Mediterranean, ketogenic, and polyphenol-rich diets—provide synergistic benefits through multiple mechanisms, including SCFA enhancement and antioxidant protection; pharmacological approaches targeting specific receptors (FXR agonists, IDO1 inhibitors) and vagal neuromodulation offer complementary mechanistic leverage [44].

## Challenges and future directions

**Fig. 5:**
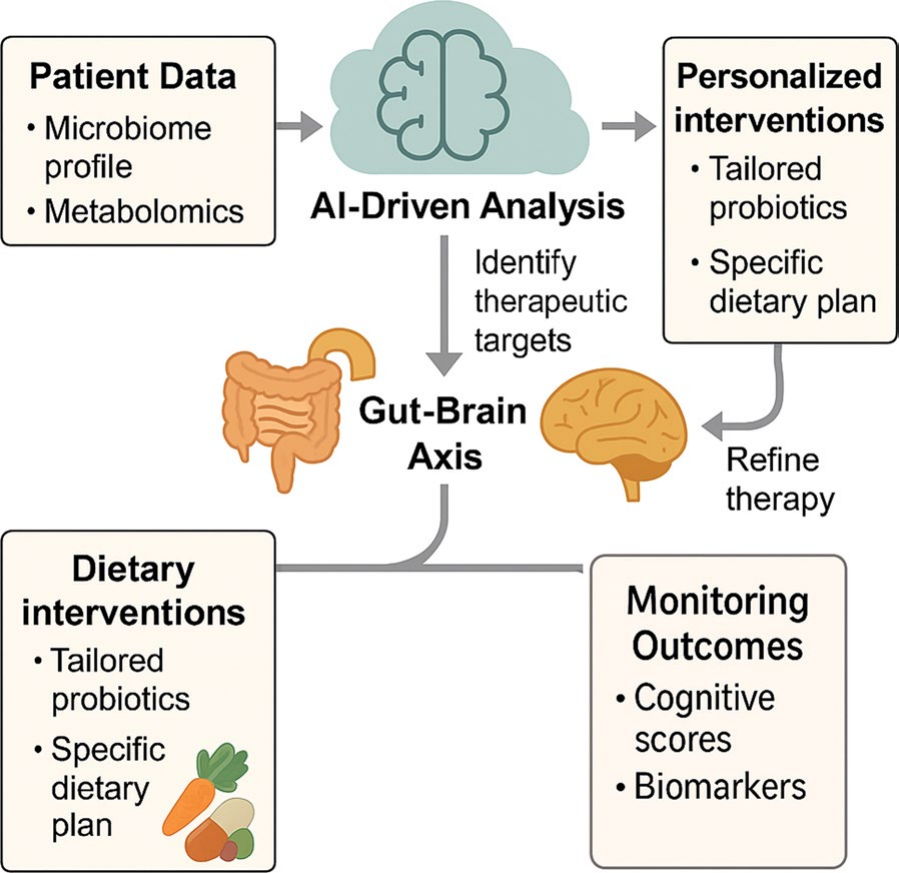
Precision medicine framework for GBA therapies

Looking to the future, a precision medicine approach (Fig. 5) promises to tailor GBA modulation to each patient’s unique microbial and metabolic signature and close the loop through real-time monitoring of outcomes.

This figure shows a personal treatment model for AD using GBA changes. Patient data—such as microbiome profiles, metabolomics, and genomics—enters an AI analysis system that identifies therapy targets. These findings help inform custom plans, such as specific probiotics and diets. Healing outcomes are monitored through cognitive tests and biomarker tracking, creating a feedback loop that allows step-by-step improvement of treatment plans. This setup highlights data-led, evolving, and patient-focused approaches at the forefront of AD precision medicine.

### Methodological challenges

Currently, research on the gut microbiome in the context of AD is limited by methodological heterogeneity, which hinders data comparability and clinical translation. Discrepancies across sequencing platforms (16S rRNA or metagenomics), sample storage, and bioinformatics pipelines often result in conflicting microbial profiles across cohorts [160]. For example, discrepant results regarding the abundance of Bacteroides in patients with Alzheimer’s are often due to region-specific DNA extraction methods or inconsistencies at the database level [25]. Clinical trials on probiotics and FMT also suffer from inconsistencies in protocol—donor variability, dosing differences, administration routes, and duration- which affect reproducibility and the possibility of meta-analytical synthesis [161]. This field urgently needs a harmonized methodological framework. Beyond these methodological and technical obstacles lie fundamental gaps in our understanding of GBA biology in AD. Addressing these gaps is essential not only for refining experimental approaches but also for identifying the most promising therapeutic targets and understanding why certain interventions succeed or fail.

### Knowledge gaps

Dysbiosis of the gut typically shows a strong association with AD pathology in short-term animal studies. However, long-term human data are lacking, and critical questions about causality remain. In particular, it is not yet clear whether the infection process alters the microbiota, leads to metabolic changes that alter the amyloid-beta and tau pathways, or what chronic cognitive effects such microbiome-based therapies would have. One under-researched area where such gender differences should be considered—although female mice often show a more pronounced response to probiotics, possibly due to hormonal modulation of microglial priming and overall immune responses—is that future human studies should consider gender as an important variable [162]. Recognizing these challenges and knowledge gaps enables us to strategically prioritize future research directions that will accelerate the translation of GBA science into effective AD therapeutics. The following research priorities represent consensus areas where investment is most likely to yield transformative advances.

### Future research priorities

These proposed directions will likely advance our understanding of the GBA in AD. Large, multi-ethnic, longitudinal studies covering all age groups and integrating microbiome, metabolome, and neuroimaging data will be key to deciphering the gut-brain temporal dynamics in AD development. Mechanistic studies in humanized mouse models with appropriate CRISPR-based microbiome editing may provide powerful approaches to decipher causal relationships between specific microbial elements, their metabolic by-products, and the occurrence of neuropathology in a host. All forms of artificial intelligence and machine learning will be important for analyzing complex multi-omics data to improve patient stratification, predict treatment response, and discover new trends. As the field moves toward potential microbiome engineering strategies, developing robust ethical and regulatory frameworks will be critical to address the inherent risks, particularly among vulnerable populations affected by cognitive decline. While CRISPR-Cas9 for targeted bacterial gene manipulation holds therapeutic potential in areas such as LPS synthesis and amyloid clearance, it requires a thorough assessment of its safety profile, potential off-target effects, and overall impact on the fragile microbial ecosystem [160]. Sect. “Challenges and future directions” identifies key challenges and future directions needed to translate GBA research into effective Alzheimer’s disease therapeutics. Methodological issues—such as lack of standardization in microbiome profiling, insufficient longitudinal studies, and varied intervention protocols—currently hinder reproducibility and comparability across studies [17]. Knowledge gaps remain regarding causality versus correlation, optimal timing of interventions throughout the AD continuum, mechanisms of individual response variability, and the long-term safety of microbiome manipulations.

## Conclusion

The GBA represents a therapeutically actionable target in the pathogenesis of Alzheimer’s disease, with gut microbiota dysbiosis serving as both an early diagnostic indicator and a modifiable risk factor. Emerging evidence supports the use of gut-derived metabolites—including SCFA ratios, indole-3-propionic acid, and TMAO—as blood-based biomarkers for detecting prodromal AD and for risk stratification, providing non-invasive alternatives to traditional CSF or neuroimaging markers [44, 163]. Key translational opportunities include: (1) precision microbiome therapeutics tailored to baseline microbiota composition, APOE genotype, and metabolic phenotypes, incorporating strain-specific probiotics (such as Clostridium butyricum and Bifidobacterium longum), targeted prebiotics, and optimized FMT protocols; (2) pharmacological modulation of metabolite-receptor pathways (including FXR agonists, AhR modulators, and IDO1 inhibitors) to normalize BA signaling, tryptophan metabolism, and neuroinflammatory cascades; and (3) multi-target combination therapies that integrate dietary polyphenols, anti-inflammatory agents, and microbiome modulators to address the multifactorial etiology of AD.

Critical gaps that require investigation include: (1) establishing causality through standardized longitudinal multi-omics studies that integrate metagenomics, metabolomics, and neuroimaging while rigorously adjusting for confounding factors (such as diet, medications, and comorbidities); (2) defining optimal FMT parameters, including donor selection criteria, engraftment kinetics, and long-term microbiome stability across diverse patient populations; (3) developing regulatory frameworks for microbiome-based diagnostics and therapeutics to ensure safety monitoring, clinical efficacy endpoints, and ethical considerations in cognitively impaired populations; and (4) elucidating individual variability in therapeutic responses through host-microbiome-drug interaction studies that incorporate pharmaco microbiomics and personalized treatment algorithms [44, 164] Collaborative, multidisciplinary initiatives to address these translational challenges will be essential for advancing GBA interventions and ultimately realizing their therapeutic potential in AD prevention and treatment.

## Data Availability

No datasets were generated or analysed during the current study.
